# Polymeric Nanoparticles for Antimicrobial Therapies: An up-to-date Overview

**DOI:** 10.3390/polym13050724

**Published:** 2021-02-27

**Authors:** Vera Alexandra Spirescu, Cristina Chircov, Alexandru Mihai Grumezescu, Ecaterina Andronescu

**Affiliations:** 1 Department of Science and Engineering of Oxide Materials and Nanomaterials, University Politehnica of Bucharest, 011061 Bucharest, Romania; veraspirescu@gmail.com (V.A.S.); cristina.chircov@yahoo.com (C.C.); ecaterina.andronescu@upb.ro (E.A.); 2 Research Institute of the University of Bucharest—ICUB, University of Bucharest, 050657 Bucharest, Romania

**Keywords:** polymeric nanoparticles, antimicrobial therapy, medical field, antimicrobial resistance, toxicity, limited patient compliance, nanotechnology, nanomaterials, up-to-date overview, targeting strategies

## Abstract

Despite the many advancements in the pharmaceutical and medical fields and the development of numerous antimicrobial drugs aimed to suppress and destroy pathogenic microorganisms, infectious diseases still represent a major health threat affecting millions of lives daily. In addition to the limitations of antimicrobial drugs associated with low transportation rate, water solubility, oral bioavailability and stability, inefficient drug targeting, considerable toxicity, and limited patient compliance, the major cause for their inefficiency is the antimicrobial resistance of microorganisms. In this context, the risk of a pre-antibiotic era is a real possibility. For this reason, the research focus has shifted toward the discovery and development of novel and alternative antimicrobial agents that could overcome the challenges associated with conventional drugs. Nanotechnology is a possible alternative, as there is significant evidence of the broad-spectrum antimicrobial activity of nanomaterials and nanoparticles in particular. Moreover, owing to their considerable advantages regarding their efficient cargo dissolving, entrapment, encapsulation, or surface attachment, the possibility of forming antimicrobial groups for specific targeting and destruction, biocompatibility and biodegradability, low toxicity, and synergistic therapy, polymeric nanoparticles have received considerable attention as potential antimicrobial drug delivery agents. In this context, the aim of this paper is to provide an up-to-date overview of the most recent studies investigating polymeric nanoparticles designed for antimicrobial therapies, describing both their targeting strategies and their effects.

## 1. Introduction

Microorganisms are an essential part of human existence, being responsible for numerous and diverse processes, including nitrogen fixation, vitamin production, photosynthesis, and organic matter decomposition. However, the delicate balance between microorganisms and the immune system may shift in favor of microorganisms, thus causing immune deficiencies [1]. Therefore, diseases caused by pathogenic microorganisms, such as bacteria, viruses, fungi, parasites, protozoa, or algae, can be directly or indirectly (vector-borne) transmitted from one individual to another, which is termed as infectious diseases [2,3,4,5].

In antiquity, around half of the individuals died before reaching sexual maturity, while in late medieval times, one-third of babies died in their infancy, which was mostly due to infectious diseases [6]. Thus, the discovery of antimicrobials, including antibacterial, antiviral, antifungal, and antiparasitic or anthelmintic drugs, has greatly impacted the global health system, as the infection-related mortality was significantly reduced [4,7,8]. Specifically, they have allowed the early treatment of infections without identifying the pathogen, consequently bringing novel possibilities for modern medicine, such as surgery, cancer chemotherapy, organ transplantation, and premature infant care [9]. While considerably numerous antimicrobials have been developed for the suppression and destruction of pathogenic microorganisms, infectious diseases are still one of the major worldwide causes of death for both adults and children, affecting millions of lives daily [10,11]. Moreover, the Sustainable Development Goals, created in 2015, classified infectious diseases as a priority for health policies [12].

The inefficiency of antimicrobials is mainly associated with their low transportation rate across cellular membranes and low activity inside the cells, which lead to limited inhibitory, cidal, and static effects on microorganisms [10]. Additionally, antimicrobials are associated with low water solubility, oral bioavailability and stability, inadequate drug targeting, non-negligible toxicity, and limited patient compliance due to frequent drug administration requirements [13]. Another major challenge is that the antimicrobial resistance of microorganisms resulted from the overuse and abuse of antimicrobial drugs, which has become a critical and serious health problem [7,10,14,15]. The World Health Organization (WHO) has warned about the real possibility of a pre-antibiotic era, identifying 12 emerging superbugs resistant to many antibiotics [9,16,17,18]. For instance, about 40% of the *Staphylococcus aureus* strains present within hospitals are resistant to methicillin, accounting for almost 120,000 blood-borne infections and 20,000 related deaths in the United States in 2017 [4,13]. Contributing to approximately 700,000 deaths annually across the world, it is estimated that by 2050, antimicrobial resistance will affect 230 million people and result in 10 million deaths annually [4,9,13,19,20]. As the discovery of novel classes of antimicrobials has slowed down since 1987, the situation is drastically worsening, and the world is facing the risk of returning to the “medical dark ages” [16,17,21,22]. Therefore, there is an urgent need for the development of novel alternative approaches to tackle the antimicrobial-resistant pathogen crisis [7,9,10,13,20,23].

**Figure 1:**
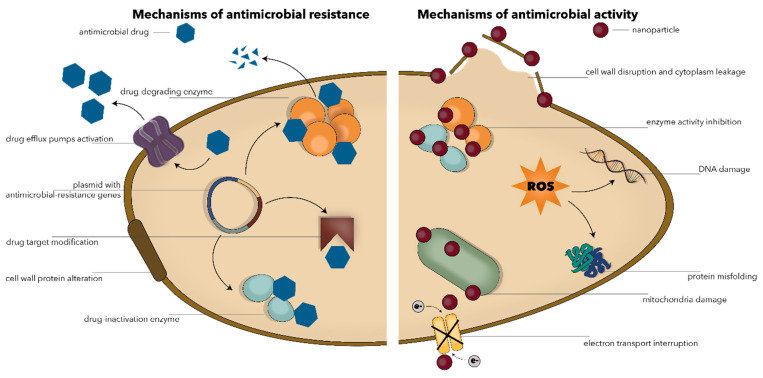
Schematic representation of the main mechanisms involved in the antimicrobial resistance (**left**) and the antimicrobial activity of nanoparticles (**right**).

The recent advancements in nanotechnology have provided a new means for improving the efficiency of antimicrobial therapies [11]. Nanomaterials and nanoparticles in particular have proven a broad spectrum of antimicrobial activity against Gram-negative and Gram-positive bacteria, mycobacteria, viruses, fungi, bacteriophages, protozoa, and algae [24,25,26]. The two main strategies for using nanoparticles as antimicrobial agents involve combatting antimicrobial drug resistance themselves or acting as carriers for the delivery of conventional antimicrobials [24,27,28]. Specifically, while the precise mechanisms are not completely understood, it has been demonstrated that nanoparticles can penetrate and disrupt the microbial cell membrane through membrane-damaging abrasiveness, induce intracellular antimicrobial effects such as the production of reactive oxygen species, interact with DNA/RNA and proteins, inactivate enzymes, increase efflux by overexpressing efflux pumps, decrease cell permeability, release metal ions, and hinder biofilm formation (Figure 1) [24,25,27,29,30,31]. The antimicrobial activity of nanoparticles is directly affected by variables such as chemistry, particle size and shape, surface-to-volume ratio, and zeta potential [17].

Recent years have witnessed increasing attention toward nanostructured antimicrobial polymers owing to their superior advantages, i.e., efficient cargo dissolving, entrapment, encapsulation, or surface attachment, the possibility of forming antimicrobial groups for specific targeting and destruction, biocompatibility and biodegradability, low toxicity, and synergistic therapy [17,32]. In this context, the aim of this paper is to provide an up-to-date overview of the most recent studies investigating polymeric nanoparticles designed for antimicrobial therapies, describing both their targeting strategies and their effects.

## 2. Microbial Targeting Strategies

Generally, the design and development of nanosized systems for the delivery of antimicrobial drugs must consider the fulfillment of several characteristics, namely improving the efficiency of the antimicrobial treatment, increasing the local concentration of the antimicrobial drug at the specific infection site, minimizing the accumulation of the antimicrobial drug within healthy tissues, and the exposure of the commensal microflora to sub-lethal doses for avoiding the development of antimicrobial resistance and reducing the associated risks of toxicity [33]. In this context, nanoparticles must be specifically designed and/or improved to achieve an efficient payload binding capacity, targeted delivery, selective recognition, and increased cellular uptake and internalization with no cytotoxicity for the host cells [34,35,36,37,38].

**Figure 2:**
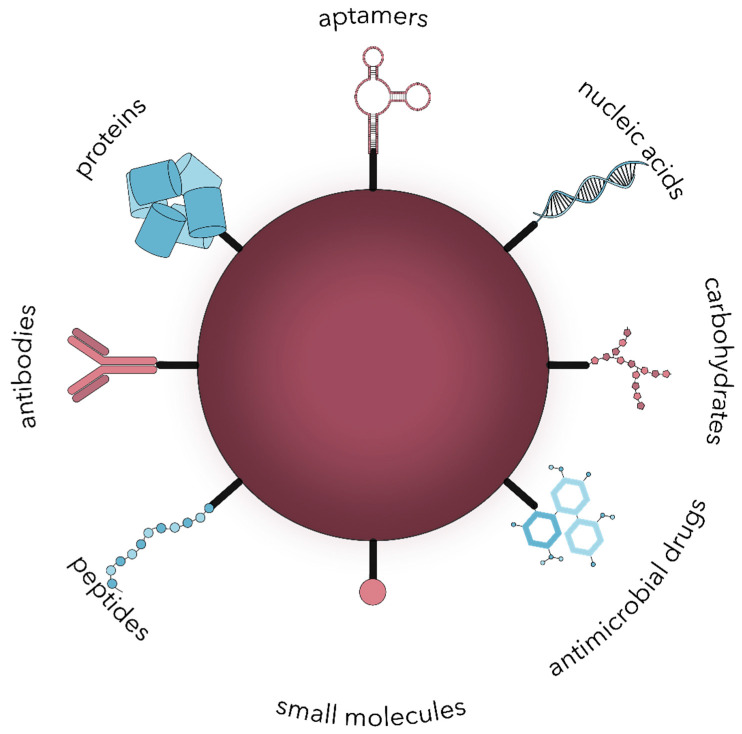
The main types of biomolecules used for the surface modification of nanoparticles for active microbial targeting.

Nanoparticles can target microbial cells through two general possibilities, namely passive or active targeting [39]. On one hand, passive targeting is associated with the accumulation of nanoparticles at the infection site due to a higher vascular permeability and impaired lymphatic system functioning, leading to prolonged drug retention [39,40,41]. This type of delivery is directly influenced by several factors, including hydrophobicity, van der Waals forces, and static electric attraction. Its potency can be increased with the electrostatic interactions between the negative charge of the bacteria surface and the cationic charge of the nanoparticle surface [40,42]. On the other hand, active targeting is a widely employed phenomenon based on the conjugation of active molecules onto the surface of nanoparticles that will specifically bind to the surface molecules overexpressed by microbial cells, such as polysaccharides, proteins, or lipids [39,40,43,44,45]. In this manner, a wide variety of functional groups and molecules or stimuli-responsive ligands can be attached through chemical, physical, or biological methods [35]. Active targeting is an advantageous method to improve the therapeutic index by increasing the selectivity and recognition properties of nanoparticles and consequently reaching higher concentrations at the specific infection site in shorter periods. Thereby, side effects and their associated socio-economic costs are considerably reduced [34,35,43,44]. Nonetheless, active targeting nanoparticles’ design should ensure an appropriate balance between the interaction strength, the release rate, and the conjugate stability [39]. Common biomolecules used as moieties for active targeting include small molecules, peptides, antibodies, nanobodies, proteins, nucleic acids, carbohydrates, and antimicrobial drugs (Figure 2) [34,35,36,39,40,41,43,46,47,48].

### 2.1. Small Molecules

The use of small molecules for the surface functionalization of nanomaterials has brought a new perspective for biomedical applications, as they possess the ability to modulate the biological properties of nanomaterials. In this manner, binding to the host cell receptors can be mediated, and antimicrobial resistance can be further avoided [40].

One example of such molecules is antifolates, with folic acid as the central molecule of their metabolism [49]. Folic acid or folate is a group of water-soluble metabolites that belong to the B-vitamin family, namely B_9_. Folates function as enzymatic co-factors in the C1 transfer reactions, playing fundamental roles in amino acid metabolism and purines, pyrimidines, and methionine synthesis. Alternatively termed tetrahydrofolate molecules, they are characterized by a common chemical structure comprising a pteridine ring, a *p*-aminobenzoic acid, and one or more γ-linked L-glutamate residues [49,50,51]. By contrast to bacteria, fungi, and some plants, mammalian cells are unable to synthesize folate de novo, but they possess specific folate receptors that allow for its internalization. As microbial infections are characterized by an accelerated folate metabolism rate in order to ensure the proper functioning of the processes involved in cell replication and protein and nucleic acid synthesis, folic acid antagonists have been widely used as alternative antibiotic agents [49,50,51,52]. Moreover, antifolates such as pyrimethamine, proguanil, and sulfadoxine represent an important class of antimalarial drugs [53,54]. Additionally, the specific cyclosporiasis drug treatment is based on the combination of two antibiotics, namely trimethoprim and sulfamethoxazole or co-trimoxazole. Their action involves blocking two consecutive steps required for the biosynthesis of nucleic acids and proteins essential for the parasite by inhibiting tetrahydrofolic acid and dihydrofolic acid production, respectively [55].

### 2.2. Peptides

The pathway involved in the targeted drug release using nanoparticles aims to accomplish the complex tasks of specifically targeting the cells of interest, crossing the extracellular membrane and internalization into the cell, and localization to specific subcellular organelles. In this context, peptides have been extensively researched and exploited for their capacity to fulfill these aims after their surface attachment onto the surface of the nanoparticles [56,57].

Therefore, antimicrobial peptides have received a great interest in naturally occurring antimicrobials produced by bacteria, fungi, protozoa, and some plants and animals, with an improved biocompatibility, selectivity, and efficiency, safety, and tolerance by the human organism [40,58,59,60,61]. These peptides comprise 5–50 amino acid chains and are generally composed of L-amino acids, such as lysine, arginine, and histidine, which are defined in α-helices and/or β-sheets secondary structures [60,62]. Their amphipathic or cationic structure allows for an efficient microbial membrane targeting and a broad action spectrum against Gram-positive and Gram-negative bacteria, fungi, viruses, and protozoa [58,60]. Owing to their biochemical properties, the action mechanisms are generally based on their interaction with the microbial phospholipid membranes [60]. Specifically, subsequent to the antimicrobial peptide binding to the target membrane, permeabilization of the membrane occurs, consequently causing cellular component leakage and cell death [63]. This binding is a result of the electrostatic forces between the positively charged antimicrobial peptides, i.e., an overall charge of +1 to +7, and the negatively charged microbial membrane. Moreover, peptide features such as the amino acid sequence, the amphipathic charge, the structure, and the hydrophobicity are key factors in the overall antimicrobial activity. Therefore, the active targeting can be modulated, as antimicrobial peptides have a tendency of binding to microbial cells, rather than mammalian cells, which are zwitterionic. Furthermore, studies have shown that with sufficient positive charges present, reducing their hydrophobicity favors bacterial cell targeting [62].

The models proposed for membrane permeabilization involve (i) the toroidal-pore model based on the accumulation of peptides on the surface, which leads to a continuous lipid monolayer bending through the pore and their building up within the pore, (ii) the barrel-stave model, through which the hydrophilic part of the peptide moves from the core interior region, while the hydrophobic part is localized toward the lipids, thus inserting into the cell membrane, and (iii) the carpet-like model, where the peptides parallelly aggregate to the microbial membrane and cover it without forming any pores, and membrane permeabilization is triggered by peptides attached to the surface, thus causing its disruption in a manner similar to detergent mechanisms that result in micelle formation [63].

### 2.3. Proteins

Nanoparticle functions and targeting can also be enhanced by protein coating [35,64]. The lysozyme is a potential candidate, as it is a biomolecule with key defensive roles in the innate immune system widely distributed in phages, bacteria, plants, vertebrates, and humans [65]. Owing to its antibacterial, antiviral, and anti-inflammatory properties, it has been extensively used in the medical, environmental protection, and food industries [35,65,66,67]. The lysozyme’s antimicrobial activity mainly relies on the degradation of the microbial cell wall through the peptidoglycan hydrolyzation, specifically the β-1,4-glycosidic bonds between N-acetylmuramic acid and N-acetylglucosamine monosaccharides [35,65,66,67,68]. Furthermore, there is evidence of lysozymal antiviral character based on its potential activity against the human immunodeficiency virus [65].

The field of antimicrobial therapy has also witnessed the emergence of monoclonal antibodies that are able to target specific microbial phenotypes [69,70]. Their advantages are numerous, including but not limited to high specificity against one specific microbial type, longevity, and multiple antimicrobial mechanisms that limit toxicity and resistance [71]. An example of such types of antibodies is the human monoclonal antibody 3E9-11, which specifically targets the O25b O-antigen present within the *Escherichia coli* ST131 O25b:H4 clonal group that is associated with extended-spectrum beta-lactamases acquisition and fluoroquinolone resistance [69]. Furthermore, monoclonal and polyclonal antibodies targeting the poly-N-acetyl-d-glucosamine and the deacetylated poly-N-acetyl-d-glucosamine polysaccharides that are highly conserved and expressed by a multitude of microorganisms, including Gram-positive and Gram-negative bacteria, fungi, and protozoa, have shown promising in vitro and in vivo efficiency [72].

Nanobodies, a novel and unique class of single domain antibodies derived from naturally occurring heavy-chain-only antibodies only present within camelid serum, have been widely investigated in recent years for their superior physicochemical properties. Specifically, nanobodies are nanoscaled compounds with a robust structure, high stability, antigen-binding affinity, one cognate target specificity, water solubility, and reversible refolding that have the potential for the development of next-generation biomolecules [73,74]. These polypeptides with a molecular weight of less than 15 kDa have emerged from the phage display process and are an important tool for developing novel nanobiotechnologies, as some are already under clinical investigation for a variety of human diseases, such as chronic inflammation, brain tumors, breast cancer, lung disorders, and infectious diseases [73,74,75]. Specifically, the European Medicines Agency (EMA) and the US Food and Drug Administration (FDA) have approved the use of caplacizumab, which is a bivalent nanobody with efficiency in the treatment of thrombotic thrombocytopenic purpura [74]. Nanobodies have also proven their efficacy in the targeting of specific microbial strains, including bacteria, viruses, and protozoa [76,77].

### 2.4. Nucleic Acids

Nucleic acid–nanoparticle complexes have also attracted significant interest, as most of the small interfering RNA (siRNA) or microRNA delivery systems have been approved for clinical trials for virus infections, cancer therapy, and many other diseases [78].

Moreover, aptamers are a special kind of targeting biomolecules, possessing excellent physicochemical features for superior infectious disease diagnosis and treatment [75]. Precisely, they are short single-stranded nucleic acids or peptides with defined three-dimensional structures and the ability to recognize and bind targets with high affinity and specificity [75,79,80,81,82]. Aptamers can discriminate between molecules structurally different with only one group or even enantiomers, establishing dissociation constants in the picomolar to nanomolar range for high molecular weight targets nanomolar to the micromolar range for low molecular weight targets [81]. They are generally obtained through a process of in vitro selection, following the methodology described by Tuerk and Gold in 1990 of The Systematic Evolution of Ligands by Exponential Enrichment (SELEX) [75,79,80,81,82]. The SELEX scheme involves three main steps, namely library and target incubation, aptamer–target complexes separation from unbound oligonucleotides, and bound molecules amplification [83]. Their small size, low molecular weight, stability under a variety of conditions, and lack of toxicity have allowed for an efficient nanoparticle functionalization for numerous active targeted delivery applications [79,80,81]. Moreover, they are a potential alternative for the use of antibodies, as they are able to overcome their limitations, such as higher temperature stability [80]. Therefore, aptamer–nanoparticle systems’ potential relies on the increase of targeting interactions due to a higher aptamer density onto the surface of the nanoparticles while being protected from the nuclease digestion [81].

### 2.5. Carbohydrates

Carbohydrates are an essential type of macromolecules that are ubiquitously found throughout living organisms, playing vital roles in numerous biological recognition processes associated with cell differentiation, development, adhesion, communication, and signaling [84]. In the context of infectious diseases, many pathogenic microorganisms attach onto the surface of host cells through carbohydrate–protein interactions between cell-surface glycans and adhesins or agglutinins. Bacteria, viruses, and fungi express onto their surface a vast number of glycan-binding proteins, which are also known as lectins [84,85,86].

The sugar-binding activity of lectins has led to various in vivo functions, including host–pathogen interactions, nutrition absorption inhibition, intercellular recognition and signal transduction, and cell migration [87,88]. In this manner, glycans could represent an important candidate for nanoparticle surface functionalization for the active targeting of microorganisms. There are many studies proving the potential of glycoprotein binding of many viruses, including human immunodeficiency virus-1, influenza, coronavirus, Ebola, Zika, herpes simplex, etc. [89], and bacteria, e.g., *Escherichia coli*, through the interaction between gluconamide-functionalized nanoparticles and the lipopolysaccharide molecules present onto the outer membrane of the microorganisms [90].

### 2.6. Antimicrobial Drugs

Nonetheless, the surface of nanoparticles can also be modified using vancomycin, which acts by targeting peptidoglycans present onto the surface of Gram-positive bacteria, polymyxin, which is responsible for targeting lipopolysaccharides found onto Gram-negative bacteria, or zinc(II)-bis(dipicolylamine), which targets phosphatidylserine and is present onto the surface of both Gram-positive and Gram-negative bacteria. Notably, they are able to specifically bind bacteria, since healthy mammalian cells do not express these types of molecules [45].

Vancomycin is a broad-spectrum glycopeptide antibiotic that specifically binds Gram-positive bacteria, including staphylococci, streptococci, and most enterococci, through hydrogen bonds between its carbonyl and amine groups and the peptidoglycans found onto the cell wall [91,92,93]. The mechanisms of action mainly involve the inhibition of the cell wall synthesis by forming non-covalent complexes with the C-terminal L-Lys-D-Ala-D-Ala motif within the bacterial peptidoglycan precursors and the inhibition of RNA synthesis [92,93,94]. While the administration of vancomycin has resulted in the development of vancomycin-resistant *Staphylococcus aureus*, vancomycin-intermediate *Staphylococcus aureus*, and vancomycin-resistant enterococci [92], its attachment onto the surface of nanoparticles has allowed for the capture of both Gram-positive and Gram-negative bacteria within complex samples, such as urine or blood [91].

Polymyxins are a class of cationic polypeptide antibiotics comprising five types of compounds, namely polymyxin A-E, which is the standard gold treatment against Gram-negative bacterial infections [95,96,97]. While their use has been avoided in the 1970s and 1980s due to the introduction of presumably safer broad-spectrum antibiotics, the emergence of multiple drug-resistant Gram-negative bacteria, especially *Pseudomonas aeruginosa* and *Acinetobacter baumannii*, has led to its clinical reintroduction [96,98]. The precise mechanisms involve binding to the bacterial cell wall and subsequently altering the outer and inner membrane permeability to K^+^ and Na^+^ ions. In this manner, the osmotic barrier of the cell is lost, leading to the death of the bacterium through lysis [8].

### 2.7. Stimuli-Responsive Nanosystems

**Figure 3:**
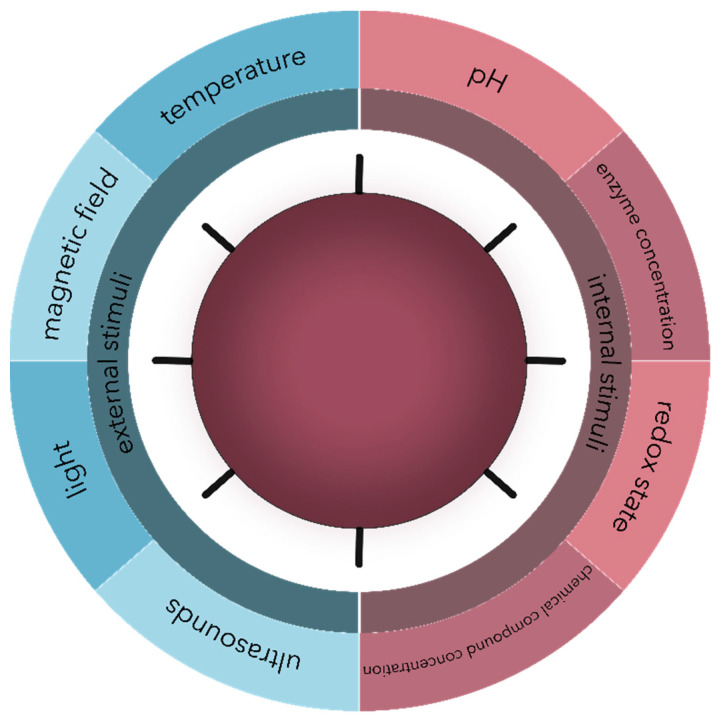
The main types of external and internal stimuli are involved in the controlled release of drug delivery nanosystems.

Another approach for microbial targeting is the development of stimuli-responsive nanosystems, which can either recognize specific microenvironmental changes associated with the pathological state of infection or inflammation, such as pH, enzyme, and chemical compound concentrations, and redox state variations, or respond to external physical stimuli, such as thermal, magnetic, light, or ultrasound effects (Figure 3). Consequently, the nanosystems react dynamically, leading to a controlled release of the drug at the targeted site [33,44,99,100]. Similarly, the targeting characteristics and the efficiency of the antimicrobial therapy are considerably enhanced, while the side effects are significantly minimized [33,99]. Additionally, this approach allows for the reversibility to the nanosystems’ initial state to control the antimicrobial effects [33,44].

An example of such nanosystems involved developing gentamicin sulfate-functionalized nanoparticles covalently grafted onto the surface of titanium implants. As the bioactive molecules were linked to the nanoparticles through pH-sensitive imine bonds, a decrease in the local pH induced by bacterial proliferation and infection would lead to the drug’s release due to the hydrolysis of the imine bond [93,101].

## 3. Antimicrobial Applications of Polymeric Nanoparticles

Due to their insolubility and administration route, bioactive compounds are generally prone to lose their pharmacological activity. Thus, the process of drug discovery and development must be optimized in order to ensure optimal pharmacokinetics, absorption, distribution, metabolism, excretion, toxicity, and therapeutic effect duration [102]. In the context of infectious diseases, the life and bioavailability of antimicrobial drugs must be enhanced, while the administered dose must be reduced [103,104].

**Figure 4:**
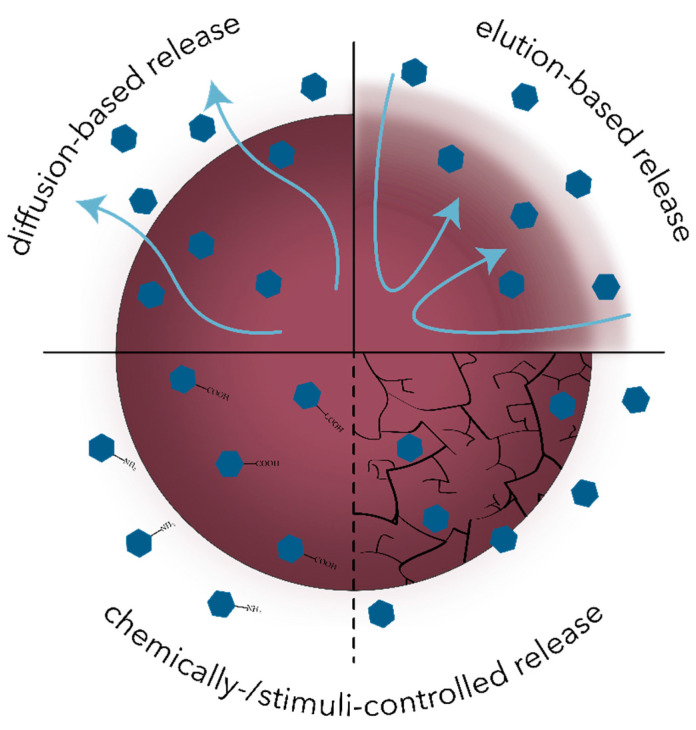
Schematic representation of the main mechanisms involved in the controlled release of antimicrobial drugs.

After administration, conventional antimicrobial drugs are distributed throughout the body via bloodstream, where a considerable percentage of the drug undergoes rapid clearance and inactivation. By contrast, drug-carrying nanosystems have the capacity to stay in the circulatory system for longer time periods and specifically target the tissue of interest. In this manner, an appropriate drug dose is administered, thus reducing the plasma fluctuations and the associated adverse effects. Furthermore, the nanoscale of these systems allows for an improved penetration through the tissue barriers while ensuring the protection of the drug until cellular uptake and targeted release [102,103,105,106]. The main mechanisms involved in the controlled release of antimicrobial drugs include diffusion-based, elution-based, and chemically- or stimuli-controlled release (Figure 4) [105].

In this context, nanostructured systems appear to be an ideal tool for combating antimicrobial resistance and developing efficient treatment options [102]. As previously mentioned, polymeric nanosystems are superior due to many advantages, such as high drug solubility and storage, biocompatibility, biodegradability, and stability, permitting the deliberate and precise drug release at the targeted sites [103,106,107].

**Figure 5:**
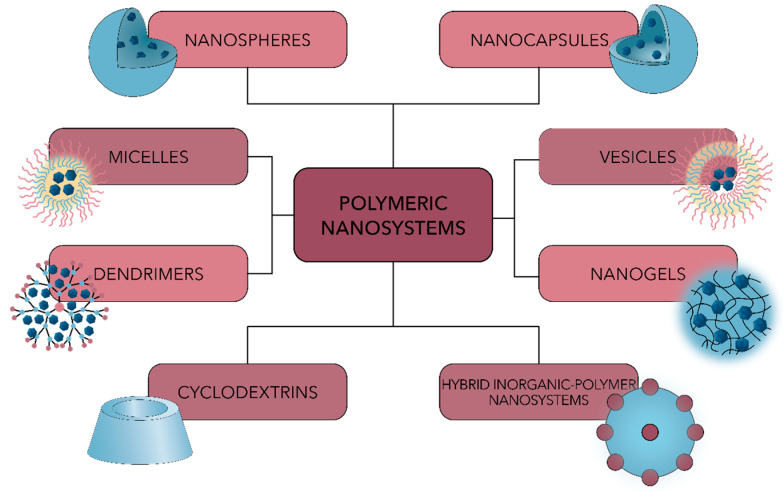
Schematic representation of the main types of polymeric nanosystems used for antimicrobial drug delivery.

Polymeric nanosystems can be synthesized from a variety of natural or synthetic precursors, such as collagen, chitosan, gelatin, or albumin, and polyethylene glycol, polylactic acid, poly(lactic-co-glycolic acid) (PLGA), polylactic acid (PLA) or polycaprolactone (PCL), respectively [42,108,109]. Additionally, they can be developed in multiple forms, including nanoparticles, micelles, vesicles, dendrimers, or hybrid inorganic–polymer nanosystems (Figure 5) [17].

**Figure 6:**
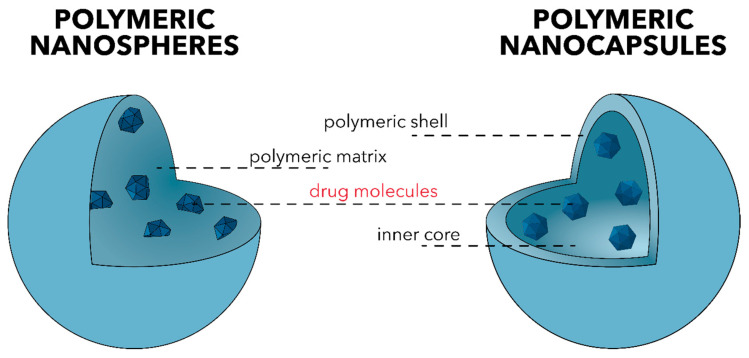
The two main types of polymeric nanoparticles—polymeric nanospheres (**left**) and polymeric nanocapsules (**right**).

This review focuses on polymeric nanoparticles, which are categorized into polymeric nanospheres and polymeric nanocapsules, depending on their internal structure and morphology (Figure 6). On one hand, nanospheres comprise a continuous polymeric network with a regular sphere structure in which drug molecules are either retained inside the matrix or attached to its surface. On the other hand, nanocapsules consist of a polymeric shell surrounding the liquid/solid oily core. The drug is dissolved and modulates the release profile of the drug [110,111].

### 3.1. Antibacterial Nanoparticles

The pathway involved in bacteria growth is based on the conversion of chemical nutrients that enter into the bacterial cell through its pores into biomass. In this manner, the biomass increase leads to increased cell size and bacterial DNA replication, and finally to the division into two daughter cells [112]. There are two types of bacterial growth, namely the planktonic growth associated with the free-swimming unicellular phase that is not attached to any surfaces, and the biofilm growth phase, which is related to the multicellular sessile state that results in community formation [47,112]. While both are a serious concern, bacterial colonization and biofilm development allow the bacteria to survive in hostile environments and form new and permanent colonies, thus posing the risk of severe systemic infections [47,113].

In this context, the studies discussed below are targeting the application of polymeric nanoparticles against both planktonic and biofilm growth. The criteria involved in the process of article selection involved papers published after 2018 from the Scopus database using the keywords “polymeric nanoparticles” and “antibacterial” or “biofilm”. Thus, 24 relevant studies were identified and categorized according to the type of polymer used, either natural or synthetic.

Table 1 summarizes all the identified studies investigating the use of natural polymers to develop nanoparticles applied in antibacterial therapies. Among natural polymers, chitosan is the most widely used as a nanocarrier to deliver both antibiotics and alternative antibacterial drugs. For instance, Qiu et al. developed phosphatidylcholine–chitosan hybrid nanoparticles coated with the gentamycin antibiotic. The reason for the introduction of the lipid component is based on the potential of lipid drug carriers to fuse with the bacterial phospholipid membrane. The results confirmed the synthesized system’s capacity to inhibit both Gram-positive and Gram-negative bacteria growth and biofilm formation [114]. Alruwaili et al. also investigated the antibacterial effects of gentamycin-containing chitosan nanoparticles that were further dispersed into pH-sensitive Carbopol polymer solutions to obtain sol–gel systems ocular delivery [115]. Ampicillin-loaded chitosan–polyanion nanoparticles were developed by Ciro et al. through ionic gelation and polyelectrolyte complexation using anionic polyelectrolytes corresponding to the sodium and potassium salts of poly(maleic acid-*alt*-ethylene) and poly(maleic acid-*alt*-octadecene) and studied for their antibacterial properties [116]. Furthermore, Evangelista et al. also synthesized supramolecular polyelectrolyte complexes based on the interactions between the positively-charged -NH_3_^+^ groups of the β-cyclodextrin-grafted chitosan and the negatively-charged -SO_3_^-^ groups of the carrageenan for antimicrobial applications. Specifically, β-cyclodextrin was used owing to its possibility to form host–guest inclusion complexes with the silver sulfadiazine molecules that will release silver ions against bacterial cells [117]. Another study by Walvekar et al. investigated the antibacterial effects of hyaluronic acid–oleylamine conjugates with different degrees of conjugation as drug nanocarriers against methicillin-resistant *S. aureus*. Precisely, the vancomycin antibiotic was encapsulated into polymersomes, which are nanocapsules comprising hydrophilic polymers grafted with long fatty acids that have the capacity to self-assemble into spherical drug carriers [118]. Moreover, Oliveira et al. developed a double-layer biomembrane comprising chitosan, hydroxypropyl methylcellulose, and lidocaine chloride as an anesthetic drug as the first layer and polymyxin B sulfate antibiotic-containing sodium alginate nanoparticles as the second layer for wound treatment [119].

By contrast, many recent studies are focusing on alternative antimicrobial agents in order to avoid the use of antibiotics that are prone to cause the resistance of the bacteria. The study performed by Ejaz et al. is an example of such applications, as they developed mannose-functionalized chitosan nanoparticles with intrinsic antibacterial properties against Gram-positive and Gram-negative bacteria and antibiofilm character [120]. Moreover, Kritchenkov et al. developed betaine-type chitosan derivative nanoparticles through an ultrasound-assisted catalyst-free thiol-yne click chemistry with antibacterial activity [121]. Alternatively, there is an increasing interest in the use of essential oils as antibacterial agents in polymer-based drug delivery systems. In this context, there are two studies performed by Hadidi et al. and Bagheri et al., respectively, that investigated the effects of clove [122] and nettle [123] essential oils encapsulated into chitosan nanoparticles. Another study by Liakos et al. investigated the antimicrobial properties of peppermint, cinnamon, and lemongrass essential oils containing cellulose acetate nanocapsules [124]. Furthermore, Ivanova et al. developed antibody-functionalized self-assembled nanocapsules comprising zein plant protein and containing oregano essential oils. This approach allows for the specific targeting of *S. aureus* bacterial strains while reducing the dosage and the system’s toxicity [125]. Other bioactive compounds that can be used as antimicrobial agents include antimicrobial glycolipids, such as sophorolipids and rhamnolipids encapsulated into chitosan nanoparticles [126], and antimicrobial peptides, such as the SET-M33 peptide, encapsulated into dextran nanoparticles [127].

Another strategy for antibacterial therapies involves developing polymeric nanoparticles as nanocarriers of both antibiotic drugs and alternative biocompounds. For example, de Oliveira et al. developed polymeric nanoparticles consisting of chitosan and hydroxypropylmethylcellulose to administer the ceftriaxone antibiotic and *S. brasiliensis* extract in antibacterial therapies [128].

Synthetic polymers are also widely used in nanoparticle development for biomedical applications [129]. In this context, Table 2 summarizes all the identified studies investigating their use in antibacterial therapies. Among them, the most commonly used include PLGA, PEG, PLA, and PCL, which have been investigated for the delivery of both antibiotics and alternative antibacterial drugs.

For instance, Ucak et al. investigated the antibacterial effect of teicoplanin-containing PLGA nanoparticles functionalized with *S. aureus*-specific aptamers [130]. Moreover, Deepika et al. synthesized PEG–PLGA nanoparticles for the co-delivery of rutin, a natural drug, and benzamide, a synthetic compound [131]. Alternatively, Durak et al. studied the effects of PLGA NPs containing natural biocompounds known for their antibacterial activities, namely caffeic acid and juglone [132]. Another study performed by Parmar et al. synthesized hybrid nanocomposites based on biogenic zinc oxide nanoparticles treated with A. indica leaf extract and PLGA [133].

Moreover, Da Costa et al. investigated the potential of rifampicin-containing PLA nanoparticles functionalized with poly-L-lysine. This cationic peptide could reverse the negative nanoparticle surface charge to positive, against planktonic bacteria and biofilm growth [134]. By contrast, Vrouvaki et al. developed PLA nanoparticles encapsulating the Pistacia lentiscus L. var. chia essential oil against Gram-positive and Gram-negative bacteria [135].

PCL was also studied for its potential in polymeric drug delivery systems applications in antibacterial therapies. Specifically, Srisang et al. prepared chlorhexidine-loaded PCL nanospheres for coating urinary catheters using a semi-automatic spray coater. The antibacterial effects of the coating were tested against common uropathogens causing urinary tract infections [136].

Other polymeric nanoparticles include cationic acrylate copolyvidone–iodine nanoparticles with a dual antibacterial activity center comprising the small molecule iodine and quaternary ammonium salt copolymers [137] and polyelectrolyte complex nanoparticles formed between the polymeric salts derived from Eudragit-E100™ and sodium salt of poly(maleic acid-alt-octadecene) for the delivery of ampicillin [138].

It can be observed that all the polymeric nanoparticles-based drug delivery systems exceed 100 nm, which is the generally accepted size limit for nanomaterials. However, considering the significant difficulty associated with the synthesis of small size polymeric nanoparticles, they are generally expected to be smaller than 200 nm in drug delivery applications in order to avoid any adverse reactions to the organism, such as embolisms. Therefore, most of the presented studies describe polymeric nanoparticles that are safe to use as nanocarriers for antimicrobial agents.

Furthermore, zeta potential was considered as the primary indicator for colloidal stability, with an appropriate stability for values other than −30 to +30 mV. Additionally, as it was previously mentioned, particles with positive surface charges exhibit increased antimicrobial potential due to the electrostatic interactions with the negative charge of the bacteria surface. Therefore, the optimum zeta potential values for polymeric nanoparticles for antimicrobial therapies should be higher than +30 mV, ensuring both the stability and the antimicrobial effects of the nanoparticles.

In this manner, chitosan remains a promising candidate for the development of antimicrobial agents due to its positively charged surface, providing intrinsic antimicrobial properties, and the possibility to synthetize nanoparticles with sizes lower than 200 nm. Additionally, chitosan is a natural polymer, thus eliminating the risk of toxicity when introduced into the organism and ensuring a proper biodegradability for a controlled drug release without toxic by-products.

Other applications involve the development of nanocoatings containing both inorganic and polymeric nanoparticles through various methods, such as the matrix-assisted pulsed laser evaporation. Examples of such studies include silver nanoparticles/PLA nanocoatings [139] or silver nanoparticles/polyethylene terephthalate nanofibers [140] and simple [141] or functionalized with lincomycin [142], cefepime [143], or *Nigella sativa* essential oils [144] magnetite nanoparticles/PLGA nanocoatings.

### 3.2. Antiviral Nanoparticles

In the context of antiviral properties, the studies discussed below are targeting the application of polymeric nanoparticles for antiviral therapies. The criteria involved in the process of article selection involved papers published after 2018 from the Scopus database using the keywords “polymeric nanoparticles” and “antiviral”. Thus, two relevant studies were identified and briefly described.

Alamdaran et al. developed chitosan nanoparticles with HIV-1 P24 protein-derived peptides adsorbed onto the surface as an alternative to counteract microbial resistance. The nanoparticles’ loading and releasing efficiency were investigated on human peripheral blood lymphocyte cells, and results showed reduced toxicity and side effects and a controlled and sustained peptide drug release [145]. Furthermore, Belgamwar et al. investigated the efficiency of dolutegravir sodium-loaded nanoparticles comprising hydroxypropyl-β-cyclodextrin cross-linked with diphenyl carbonate in order to enhance the central nervous system uptake through the intranasal administration route. Results proved an improved permeation of the drug through the nasal mucosa and access in the cerebrospinal fluid without damaging the mucosa [146].

### 3.3. Antifungal Nanoparticles

In the context of antifungal properties, the studies discussed below are targeting the application of polymeric nanoparticles for antifungal therapies. The criteria involved in the process of article selection involved papers published after 2018 from the Scopus database using the keywords “polymeric nanoparticles” and “antifungal”. Thus, five relevant studies were identified and briefly described.

Costa et al. developed chitosan nanoparticles for the co-administration of miconazole and farnesol for the treatment of vulvovaginal candidiasis, which is mainly caused by the opportunistic fungal strain C. albicans. Results regarding the microorganism growth inhibition on the C. albicans (ATCC28367) showed a minimum inhibitory concentration (MIC) for the nanosystems similar to the values for the miconazole free drug. Moreover, the nanosystems administered in the murine model of vulvovaginal candidiasis were considered the most effective for infection inhibition [147]. Similarly, Charanteja Reddy et al. developed chitosan nanoparticles incorporating itraconazole that could be potentially used against C. neoformans, C. albicans, and A. fumigatus [148]. Furthermore, nanocapsules based on Sterculia striata polysaccharide modified with propionic anhydride through the acylation reaction were synthesized by Sombra et al. for the delivery of amphotericin B. The nanosystems revealed antifungal activity against four C. albicans strains, with MIC values lower for two strains and higher for the other strains when compared to the free drug [149]. Additionally, the previously described studies by Liakos et al. [124] and Srisang et al. [136] also exhibited antifungal activity against C. albicans strains.

### 3.4. Antiparasitic Nanoparticles

In the context of antiparasitic properties, the studies discussed below are targeting the application of polymeric nanoparticles for treatment against parasitic infections. The criteria involved in the process of article selection involved papers published after 2018 from the Scopus database using the keywords “polymeric nanoparticles” and “antiparasitic”. Thus, two relevant studies were identified and briefly described.

Specifically, Real et al. studied the effect of chitosan nanocapsules containing triclabendazole, a poorly water-soluble compound used as the drug of choice in the treatment of fascioliasis. Results showed increased stability of over one month and strong interactions with enterocytes, thus enabling a higher uptake and sustained release of the drug [150]. Furthermore, the previously mentioned study performed by Durak et al. also exhibited antiparasitic effects against the Leishmania promastigotes protozoan parasites, with a dose-dependent antileishmanial [132].

## 4. Conclusions and Future Perspectives

The antimicrobial resistance of microorganisms that resulted from the overuse and abuse of antimicrobial drugs has become a critical and serious health problem that has led to many deaths worldwide. Nanotechnology has been applied to design alternative antimicrobial agents that could overcome the limitations of conventional drugs. Particularly, polymeric nanoparticles have been widely investigated for their potential to passively or actively target microbial strains and act both as a drug nanocarrier and as an antimicrobial agent due to intrinsic antimicrobial properties. In this context, numerous studies are investigating their application in antimicrobial therapies, but most are targeting bacterial pathogens. Therefore, there is still room for the improvement of polymeric nanoparticles for antiviral, antifungal, or antiparasitic applications, especially concerning the current COVID-19 pandemic situation that has been affected by the lack of effective antiviral agents. In this context, polymeric nanoparticles could efficiently target the coronavirus through active microbial targeting strategies and release appropriate quantities of the antiviral agent to destroy the pathogen.

While polymer-based nanoparticles offer a series of advantages, such as increased biocompatibility, biodegradability, and clearance from the human organisms, there are still some limitations associated with the currently available systems that must be considered. Specifically, the size and the size distribution of polymeric nanoparticles are variables that generally pose significant challenges in the manufacturing process. In this regard, microfluidic approaches could offer a potential alternative that could lead to the development of more uniform and smaller nanoparticles. Additionally, such technologies could allow for the one-step functionalization and drug encapsulation, thus eliminating some of the reaction steps involved in the conventional synthesis and ensure the optimum stability of the nanoparticles through the possibility of modulating the surface charge.

## Figures and Tables

**Table 1:** Summary of the identified studies investigating the antibacterial properties of nanoparticles synthesized from natural polymers.

Nanoparticle Type	Size Range [nm]	Zeta Potential [mV]	Targeted Bacteria			Targeting Strategy	Antibacterial Agent	Results	Ref.
			Gram-Positive	Gram-Negative	Biofilm				
phosphatidylcholine CS NPs	137.2–231.8	−27.6 to −31.8	*L. monocytogenes*, *S. aureus*	*P. aeruginosa*, *E. coli*	*L. monocytogenes*, *P. aeruginosa*	passive	gentamycin	MIC results indicated similar antibacterial effects between the NPs and gentamycin alone; biofilm mass results showed a stronger inhibition capacity of the systems than gentamycin alone.	[114]
CS NPs and CS NPs dispersed into Carbopol sol–gel systems	135.2	+25.1	*S. aureus*	*E. coli*	-	pH-responsive	gentamycin	ZOI was higher for NPs than for the marketed Gentacin eye drop, but lower than for sol–gel systems due to a sustained drug release in both bacterial types.	[115]
CS–polyanion NPs	130.7–249.2	+39.5 to +49.2	*S. aureus* (ATCC25923, ATCC29213, and ATCC43300)	-	-	passive	ampicillin	MIC increased by 50% once the antibiotic was encapsulated into the NPs, independent of the ampicillin-resistance degree.	[116]
β-cyclodextrin-grafted CS and carrageenan SPECs	10–60	−40 to +42	*S. aureus* (ATCC25923), E. durans/hirae (SS1225/ IAL 03/10)	*K. pneumoniae* (ATCC700603), *E. coli* (ATCC25922)	-	passive	silver sulfadiazine	ZOI for the drug-loaded SPECs was similar to the ZOI for the drug alone and gentamycin alone, especially in the case of Gram-positive bacteria; MIC values for the drug-loaded SPECs were equal to the values for the drug alone and half of the values for the gentamycin alone against both S. aureus and *E. coli*.	[117]
hyaluronic acid–oleylamine polymersomes	201.4–360.9	−20.4 to −17.6	*S. aureus* and MRSA	-	-	passive	vancomycin	MIC values were considerably lower for the free gentamycin, but it lost its activity after 24 h; polymersomes were not as potent as the free vancomycin but were able to improve the antibacterial effects due to a slow and controlled release over a prolonged period of time.	[118]
Double-layer membrane comprising a sodium alginate NPs layer and a chitosan and hyaluronic acid layer	n.r.	n.r.	*S. aureus* (ATCC25923)	*P. aeruginosa* (ATCC27853)	-	passive	polymyxin B sulphate	MIC values for the NPs were lower than for the drug alone; MIC values for the biomembrane were lower than for the NPs due to the synergistic antibacterial effects of the components.	[119]
mannose-functionalized CS NPs	180	+25.4	*L. monocytogenes*, *S. aureus*	*E. coli*, *P. aeruginosa*	*L. monocytogenes*, *S. aureus*, *E. coli*, *P. aeruginosa*	mannose-binding lectins	-	mannose functionalization increased inhibited bacterial growth more significantly due to the interaction with the bacterial membrane lectins; growth inhibition was higher for Gram-negative bacteria; NPs effectively reduced the adherence of bacteria in the polystyrene adherence assay; mannose-functionalized CS NPs exhibited the highest antibiofilm potential, as compared to the simple CS NPs, especially against *E. coli* and *P. aeruginosa*.	[120]
cationic betaine CS derivatives NPs	108–807	+33.1 to +69.1	*S. aureus*	*E. coli*	-	passive	-	NPs possess higher antibacterial activity than pristine polymers; antibacterial activity is dependent upon the NPs size and the ξ-potential—smaller sizes and higher ξ-potentials leads to increased antibacterial activity.	[121]
CS NPs	223.2–444.5	+10.1 to +34.5	*L. monocytogenes*, *S. aureus*	*S. typhi*, *E. coli*	-	passive	clove EOs	the highest inhibitory activity was achieved for EOs-encapsulated NPs, as compared to the pure EOs and unloaded NPs against all bacterial strains; IH values were higher for *S. aureus* and *L. monocytogenes*; MIV values were the lowest for the EOs-encapsulated NPs against all bacterial strains.	[122]
CS NPs	208.3–369.4	+14.4 to +30.1	*L. monocytogenes*, *S. aureus*, *B. cereus*	*S. typhi*, *E. coli*	-	passive	nettle EOs	the highest inhibitory activity was achieved for EOs-encapsulated NPs, as compared to the pure EOs and unloaded NPs against all bacterial strains; IH values were higher for *S. aureus*; MIV values for the EOs-encapsulated NPs were similar to the values for the pure EOs and considerably lower than the unloaded NPs.	[123]
cellulose acetate NCs	150–200	−42 to −38	*S. aureus* (ATCC25923)	*P. aeruginosa* (ATCC25324), *E. coli* (ATCC25922)	*P. aeruginosa*, *E. coli*, *S. aureus*	passive	peppermint, cinnamon, and lemongrass EOs	the most efficient were cinnamon EOs-encapsulated NCs, with significant growth inhibition of all bacterial strains, especially *E. coli*; peppermint EOs-encapsulated NCs demonstrated a low inhibitory activity against the growth of *S. aureus* and C. albicans; lemongrass EOs-encapsulated NCs slightly inhibited the development of E.coli; *P. aeruginosa* strain revealed the highest resistance to the tested NCs; lowest MIC values were obtained for the cinnamon EOs-encapsulated NCs; most significant antibiofilm formation was observed against *S. aureus* biofilms for cinnamon EOs-encapsulated NCs.	[124]
zein protein NCs	134.9	−28.6	*S. aureus* (ATCC25923)	-	-	antibody-based targeting	oregano EOs	EOs encapsulation enhanced the antibacterial effects as compared to the pristine EOs; antibody attachment further enhanced the antibacterial activity; antibody attachment ensured a more specific activity against *S. aureus* co-cultured with the *P. aeruginosa* (ATCC10145) strain; antibody attachment inhibited *S. aureus* growth and protected human skin fibroblasts in co-culture.	[125]
CS NPs	210.0/329.6	+30.8/+37.4	*S. aureus* (ATCC25923)	-	-	rhamnolipid-based targeting	sophorolipids and rhamnolipids	significantly higher MIC values for rhamnolipid-containing NPs and sophorolipid-containing NPs compared to the levofloxacin control; lower MIC values for both glycolipid-containing NPs compared to the unloaded NPs.	[126]
dextran NPs	18	−13	-	*P. aeruginosa* (PAO1)	-	SET-M33 peptide	SET-M33 peptide	similar MIC values between the free peptide and the peptide-functionalized NPs; regrowth occurred after 24 h of exposure to the nanosystems.	[127]
CS and hydroxypropylmethylcellulose NPs	440–1660	+18.1 to +38.9	-	*E. coli* (ATCC25922), *E. coli* producing extended-spectrum beta-lactamases, carbapenemase-producing *K. pneumoniae*	-	passive	ceftriaxone and *S. brasiliensis* extract	lowest MIC values for the nanosystems compared to the ceftriaxone-containing NPs and *S. brasiliensis*-containing NPs against all strains; lowest MBC values for the nanosystems compared to the ceftriaxone-containing NPs and *S. brasiliensis*-containing NPs against all strains.	[128]

CS—chitosan; NPs—nanoparticles; MIC—minimum inhibitory concentration; ZOI—zone of inhibition; SPECs—supramolecular polyelectrolyte complexes; MRSA—methicillin-resistant *S. aureus*; n.r.—not reported; EOs—essential oils; IH—inhibitory halo; NCs—nanocapsules; MBC—minimum bactericidal concentration.

**Table 2:** Summary of the identified studies investigating the antibacterial properties of nanoparticles synthesized from synthetic polymers.

Nanoparticle Type	Size Range [nm]	Zeta Potential [mV]	Targeted Bacteria			Targeting Strategy	Antibacterial Agent	Results	Ref.
			Gram-Positive	Gram-Negative	Biofilm				
PLGA NPs	226	-29	*S. aureus* (ATCC29213, ATCC25923, ATCC43300), *B. cereus* (ATCC12228), MRSA (EGE-KK-13, EGE-KK-95)	-	-	aptamer-based targeting	teicoplanin	MIC values were considerably decreased upon the encapsulation of teicoplanin into the NPs for all bacterial strains; MIC values decreased even more after aptamer attachment for the *S. aureus* strains but considerably increased for the *B. cereus*.	[130]
PEG–PLGA NPs	260–291	−22.4 to −17.6	*S. aureus* (MTCC96)	*P. aeruginosa* (MTCC2488)	*S. aureus*, *P. aeruginosa*	passive	rutin and benzamide	MIC values decreased with the encapsulation of the drugs into the NPs when compared to either drug alone; rutin and rutin-encapsulated NPs exhibited higher MIC values than benzamide and benzamide-encapsulated NPs, respectively; biofilm inhibition analysis followed a trend similar to the MIC assay.	[131]
PLGA NPs	151.4–196.1	−25.7 to −21.2	*S. aureus*	*E. coli*	-	passive	caffeic acid and juglone	MIC values were similar or slightly lower for the drug-containing NPs; ZOI were similar or slightly lower for the drug-containing NPs.	[132]
PLGA–ZnO nanocomposites	185.7	−5.9	*S. aureus*	*E. coli*	-	passive	-	ZOI were considerably higher for the nanocomposites than for the zinc oxide NPs or the standard antibiotic; ZOI were higher against *S. aureus* due to electrostatic interactions.	[133]
PLA NPs	162	+40	*S. aureus* (SH1000)	-	*S. aureus*	poly-L-lysine attached on the surface	rifampicin	MIC values against planktonic *S. aureus* were similar for all tested systems, namely the free antibiotic, antibiotic-encapsulated NPs, and antibiotic-encapsulated NPs functionalized with poly-L-lysine; antibiofilm properties were similar for all tested systems, namely the free antibiotic, antibiotic-encapsulated NPs, and antibiotic-encapsulated NPs functionalized with poly-L-lysine; interactions between poly-L-lysine-functionalized nanoparticles are dose-dependent.	[134]
PLA NPs	239.9/286.1	−29.1/−34.5	*B. subtilis* sub. *spizizenii* (DSM-347)	*E. coli* (DSM-1103)	-	passive	*Pistacia lentiscus* L. var. *chia* EOs	MIC values for the EOs-functionalized NPs were lower than for the EOs dissolved in organic solvents but higher than for gentamycin against *E. coli* and higher than all cases for *B. subtilis*.	[135]
PCL NSs	152	−10.2	*S. aureus* (ATCC25423)	*E. coli* (ATCC25922)	-	passive	chlorhexidine	inhibition of 50% growth of the microorganisms up to 15 days.	[136]
cationic acrylate copolyvidone–iodine NPs	200	+11.7	*S. aureus*	*E. coli*	-	passive	-	no bacterial growth in the presence of the NPs due to the synergistic effects of iodine and quaternary ammonium salts; NPs maintained antibacterial effects for 11 days; growth inhibition of *S. aureus* was lower than that of *E. coli*; NPs exhibited significant dose-dependent inhibitory effects.	[137]
PEC NPs	>200	≈0/>|40|	*S. aureus* (ATCC25923, ATCC29213, ATCC43300)	-	-	passive	ampicillin	different antibacterial behaviors depending on the family of the complex.	[138]

NPs—nanoparticles; MRSA—methicillin-resistant *S. aureus*; MIC—minimum inhibitory concentration; ZOI—zone of inhibition; EOs—essential oils; NSs—nanospheres; PEC—polyelectrolyte complex.

## Data Availability

Not applicable.
